# Cost-utility analysis of an implant treatment in dentistry

**DOI:** 10.1186/s12903-021-01790-y

**Published:** 2021-09-06

**Authors:** Johana Losenická, Ondřej Gajdoš, Vojtěch Kamenský

**Affiliations:** Department of Biomedical Technology, Faculty of Biomedical Engineering, Czech Technical University in Prague, nám. Sítná 3105, 272 01 Kladno, Czech Republic

**Keywords:** Implant treatment, Implant, Fixed dental prosthesis, Cost-utility analysis, Markov models

## Abstract

**Background:**

When dealing with the replacement of one missing tooth, the patient has the option of choosing between different types of treatment interventions. Several important factors play a role in his decision-making process, including his limited financial means and his efforts to solve the problem of missing teeth as effectively as possible. The main goal of the study is the economic-clinical evaluation of implant treatment, as a surgical-prosthetic method in dentistry, in case of replacement of one missing tooth of the molar area.

**Methods:**

Cost-utility analysis from the patient's perspective is used for evaluation. The selected comparator is a purely prosthetic solution with the help of a three-unit fixed dental prosthesis. Cost-utility analysis is modelled using Markov models, which consider a 30-year time horizon.

**Results:**

Based on the results of modelling, the intervention evaluated by the patient, i.e. treatment with the help of implant-supported single crown, brings exactly 15.31 quality-adjusted prosthesis years (QAPY) after 30 years. The value of incremental cost-utility ratio amounted to USD − 1434.

**Conclusion:**

The results of the cost-utility analysis suggest that implant treatment with an implant-supported single crown is more cost-effective than treatment with the three-unit fixed dental prosthesis.

## Background

The current modernization and innovation of treatment procedures is leading to a constant increase in health care costs. Increasing quality of provided care goes hand in hand with this trend. The development of technologies and new methods of treatment does not avoid the field of dentistry either. Implantology is one of the examples of a dynamically developing field of dentistry. However, patients are generally very often faced with the decision on choosing the type of treatment, and the financial side of things play an important role in their decision, sometimes the most important. Other factors that influence their final decisions include aesthetics, durability of the treatment, the time required for treatment and also the willingness to undergo surgery.

The dental implants themselves are intended to replace a missing tooth or an entire section of teeth. Various treatment strategies are possible to use in dealing with the loss of one tooth of the molar region. Treatment with a three-unit fixed dental prosthesis (FDP), is considered a conventional treatment, i.e. standard [1, 2]. In the Czech Republic, and also in the world, however, the most common type of dental implants is an implant-supported single crown (ISC) [3].

The three-unit fixed dental prosthesis is a prosthetic replacement that is able to bridge the gap after one or more lost teeth. It is therefore a partial replacement of teeth. The standard FDP has one major drawback, and that is the need for grinding, deterioration and irreversible damage to two teeth adjacent to the gap (so-called abutment teeth) [1]. Vogel [4] in his literature review highlights this fact as a major benefit of the use of the implant. The prosthetic solution of a missing tooth in the form of the FDP is today considered inadequate by many experts due to the irreversible deterioration of abutment teeth. In this context, we often talk about harm to the patient [1]. Also, higher incidence of complications is referred in connection with the FDP.

The use of implant treatment in the case of single tooth replacement is still a controversial topic in terms of costs and benefits. There is no cost study in the Czech Republic that would accurately assess the costs and benefits of this treatment. Most published foreign studies [5–8] comparing implant treatment and FDP as part of the solution to the replacement of one missing tooth agree that implant treatment entails higher initial costs. However, success, longevity and patient satisfaction are considered more favourable. Foreign studies [1, 5, 7–13] focused on economic-clinical evaluation of dental implants very often use models with long time horizon which proves that after a longer period of time, implant treatment becomes more cost-effective compared to FDP.

Studies [14, 15] also agree that implant therapy requires more visits, a longer duration of the initial phase of treatment, while the total time spent in a chair is comparable in most studies. The aim of the study is the economic-clinical evaluation of implant treatment in dentistry in comparison with FDP. Based on the aim of the study we set null hypothesis that implantology therapy is more cost-effective compared to standard conventional therapy in the case of treatment of one tooth in the molar area of the teeth.

## Methods

The CUA is used to fulfil the goal with the help of Markov models [16]. The input data of the model were consulted with members of an expert team consisting of four dentists operating in Prague. A necessary prerequisite for the selection of dentists for an expert team was their practical experience in the field of implantology and prosthetics at least 5 years. Another requirement was a complete knowledge of the clinical issues and their operation in Prague. All of them are working in a different private practice. The expert team also included a standard patient (male, 50 years old), who was treated by another private dentist and was chosen randomly in the moment of research. The reason for including the patient in the expert group was primarily the introduction of the voice of the patient perspective. He had to be presented with a decision on the selection of a treatment variant for the replacement of one missing molar area tooth as part of a dentist's treatment. All members of the expert group were male between the ages 30 and 50 (see in Table 1). No experimental research was performed within the study, an expert team was created for the purpose of consulting the input data of the model. All addressed experts and one patient were familiar with the purpose of the research and agreed to participate in expert interviews.

**Table 1:** Description of expert team

Expert	Education	Age	Years of practice
1	Stomatology – Faculty of Medicine in Hradec Králové Charles University	35	10
2	Stomatology – First Faculty of Medicine Charles University	33	9
3	Stomatology – First Faculty of Medicine Charles University	46	17
4	Stomatology – Faculty of Medicine in Hradec Králové Charles University	50	21

### Cost-utility analysis parameters

The selected comparator is a three-unit fixed dental prosthesis. The preferred perspective is the health care payer's perspective [2]. However, in the case of an implant solution for the replacement of one tooth, this is not the most advantageous, as the participation of health insurance companies in payments for this treatment is minimal. The patient's perspective was chosen in this study. The main reason is the role of the patient in the dental care system. The patient is the one who bears the highest part of the costs in the implant-surgical process.

Both treatment strategies offer three treatment variants, which differ in their complexity, length and cost. The choice of treatment variant is always made by the dentist after evaluation of the clinical condition of the tissues and after agreement with the patient. Variant A of ISC treatment involves inserting an implant without the need for bone augmentation and providing the implant with a crown, provided there is open healing. Variant B of ISC is a solution where closed healing is assumed. Variant C of ISC requires moderate bone augmentation prior to implant placement. Variant A of FDP represents tooth replacement with the help of a fixed bridge in the case of intact abutment teeth without the need for crown augmentation and endodontic treatment. Variant B of FDP offers a similar treatment, but with the need for a crown extension of the abutment teeth. Variant C of FDP offers the need for endodontic treatment of abutment teeth.

The target population in the presented model is represented by 50 years old adult patients, who primarily address the need to replace one missing tooth in the molar area of the tooth. Patients who have their own teeth adjacent to the gap are considered. Based on the nature of the problem, the methodology used in published foreign studies of cost-effectiveness and cost-utility analysis, and the value of life expectancy in the Czech Republic, the maximum time horizon of the model of 30 years was chosen. From the data of the Czech Statistical Office (CSO) [17] for 2018, it is clear that the average life expectancy of individuals living in Prague is 78.3 years (men) and 83 years (women).

The costs and utilities in the CUA model are discounted at a corresponding discount rate beyond one year. A discount rate of 3% is considered, which is considered standard in the Czech Republic [2].

### The structure of the Markov model

**Fig. 1:**
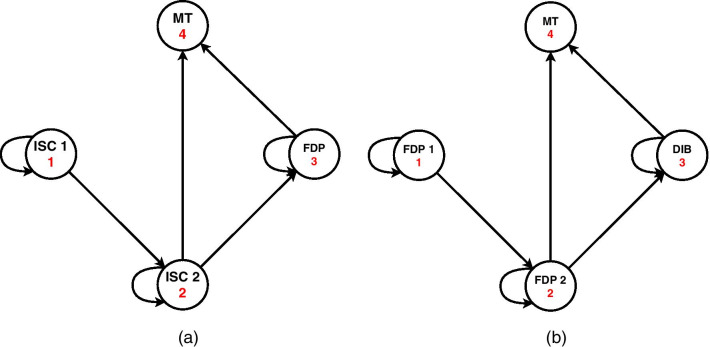
State diagram of the Markov model **a** implant-supported single crown (ISC), **b** three-unit fixed dental prosthesis (FDP). In each model, four health states are described with the possibilities of their transition. First implant-supported single crown (ISC 1), second implant-supported single crown (ISC 2), first three-unit fixed dental prosthesis (FDP; FDP 1), second three-unit fixed dental prosthesis (FDP 2), double implant-supported bridge (DIB), missing tooth (MT)

As Briggs and Sculpher [16] describe the Markov models as decision-analytical models that divide disease (process) into distinct states (see in Fig. 1). Transition probabilities describe the transition between the states over a discrete time period (cycle). Transition probabilities for our model are in Table 1 and each line represent transition probability from one state to another and the source of the information. We can assign estimates of costs and health outcomes to each the states and then calculate long-term cost and outcomes or CUA for particular healthcare intervention.

An adapted Markov model with one-year cycles was designed to calculate the CUA. The licensed TreeAge Pro [18] software, specifically its Healthcare module, was used to create the model itself. Markov's model essentially consists of two basic decision trees, each of which has its initial state as a solution to the replacement of one missing tooth, which is one of two alternatives for the treatment of a given health problem—the state "ISC" and "FDP". In the case of both treatment strategies, four health states are described in the model. State diagrams of the Markov model for an implant-supported single crown and a three-unit fixed dental prosthesis are in Fig. 1.

### Probabilities used in the model

Probabilities of mutual transitions are described between the individual health states of the model (Table 2). Missing tooth (MT) refers to a medical condition associated with a gap after a missing tooth that is not filled with a replacement. The double implant-supported bridge (DIB) is the state that is selected when a second FDP fails. DIB represents three-unit implant-supported bridge. Its incorporation into the model was inspired by a Swiss study [9] and consulted with members of the expert group.

**Table 2:** Health states of the model and values of their transition probabilities

	Transition in case of compensation failure		Data source
	To the state	With probability (%)	
Health states of ISC			
ISC 1	ISC 2	1	[5, 9]
ISC 2	FDP MT	0.998 0.002	[5]
FDP	MT	1	[5]
MT	–	–	[5]
Health states of FDP			
FDP 1	FDP 2	1	[5]
FDP 2	DIB MT	0.846 0.154	Opinion of experts
DIB	MT	1	[9]
MT	–	–	[9]

**Fig. 2:**
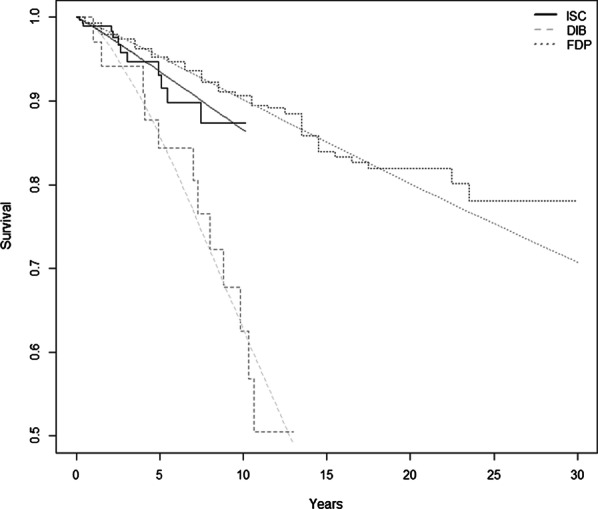
Survival curves of individual interventions. Based on the log-likelihood ratio, the Weibull probability distribution was chosen for all curves. Implant-supported single crown (ISC), three-unit fixed dental prosthesis (FDP), double implant-supported bridge (DIB)

The mentioned Markov models assume that the value of the survival rate of individual dental prostheses, as health conditions of the model, decreases with increasing number of years of action in the oral cavity. For this purpose, cumulative Kaplan–Meier survival curves of individual replacements were extracted from available foreign clinical studies [19–22], from which the probabilities of survival of replacements for individual years of the considered time horizon were derived. The most suitable probability distributions describing the survival rate were found for the relevant reconstructed Kaplan–Meier survival curves. Based on the log-likelihood ratio, the Weibull probability distribution was chosen for all curves. The interpolation of the probability of survival of ISC, FDP and DIB treatments is shown in the following figure (Fig. 2).

### Cost data collection

The costs are based on financial treatment plans and price lists of the 13 contacted private dental offices, where 4 of them represent workplaces of members of the expert team. The costs of the initial phase of treatment, both direct and indirect, as well as the costs of possible complications and the maintenance of the replacement, which represent annual costs, were considered. According to Bassi et al. [23], the indirect costs of the initial phase of treatment, which relate to the time spent by the physician and can be referred to as lost patient gain, are calculate according to formula 1.

1 $${\mathbf{Cost}}\,{\mathbf{of}}\,{\mathbf{the}}\,{\mathbf{patient}}^{\prime}{\mathbf{s}}\,{\mathbf{time}}\,{\mathbf{at}}\,{\mathbf{the}}\,{\mathbf{doctor}}^{\prime}{\mathbf{s}} = \, (1 - unemployment\,rate) \times average\,hourly\,earnings \times number\,of\,hours$$

The set weekly working hours for employees pursuant to Sect. 79 of the Labor Code [24] is 40 h per week. The Labor Code [24] also defines a coefficient of 4.348, which expresses the average number of weeks per month in an average year (365.25 days). The average gross monthly nominal wage per calculated number of employees in the national economy of the Czech Republic was overall CZK 36,144 (USD 1,726; CZK 1 = USD 0.048 as of 17^th^ May 2021), according to the CSO, in the 4th quarter of 2019. The average hourly earnings in the Czech Republic are then equal to CZK 207.82 (9.92 USD). The unemployment rate must also be included in the calculation of the so-called lost profit [7]. As of January 2020, the CSO states this at 3.1%.

The patient's transport costs to the doctor (formula 2) are processed based on the average number of visits. Data from thirteen Prague dental clinics are included in the cost analyses, therefore the amount of CZK 32 (USD 1.53) was used as a cost unit. The amount corresponds to the rate of the basic fare of Prague Public Transit Company and allows an adult to travel around Prague for 90 min.

2 $${\mathbf{The}}\,{\mathbf{patient}}^{\prime}{\mathbf{s}}\,{\mathbf{transport}}\,{\mathbf{costs}}\,{\mathbf{to}}\,{\mathbf{the}}\,{\mathbf{doctor}} = 2 - base\,fare\,rate \times number\,of\,visits\,to\,the\,doctor$$

### Collection of utility data

For the economic-clinical evaluation of ISC and FDP, it was necessary to obtain clinical results regarding both interventions. For this purpose, utility values corresponding to the health conditions presented in the model were extracted from the available studies [13, 25]. As well as costs, the utilities in the model were discounted at a 3% discount rate.

### Sensitivity analysis

A deterministic one-way sensitivity analysis was performed with the presentation of the results using graphs, tables and a tornado diagram. Selected factors that were gradually varied include initial (input) costs, utility values and the length of the time horizon (or the number of model cycles). When considering 3 cost variants (A, B, C), the standard deviation for FDP is up to 25% of the mean value, and therefore the initial (input) costs and utility values were varied by ± 30%. In the case of the length of the time horizon, these were changes in values in the interval from 5 to 55 years after five years. Subsequently, an analysis of scenarios was presented, in which there were changes in discounting. It was a scenario that does not consider the discount rate (0% discount rate) and a scenario with a 5% discount rate.

## Results

### Costs of health states

Each health state of the model was assigned the appropriate monetary units that the patient spends after finding himself in such a health state. Table 3 lists the entry costs that the patient will pay after entering the relevant health state, as well as the annual costs, which represent the amount paid by the patient each year for maintenance of the reimbursement and any complications with reimbursement if the patient remains in the relevant health state. According to study [7], the cost of DIB reimbursement is calculated as one third of the cost of FDP (considered variant A) together with twice the cost of ISC (considered variant B). The cost of entering the MT health state represents the average amount that the patient pays at the clinics for the explanation or extraction of both abutment teeth.

**Table 3:** Values of considered costs in the model

Treatment variants	Initial costs (CZK)			Annual costs (CZK)—regardless of the variant		
	A	B	C	1st and 2nd year	3rd to 5th year	6th year and more
ISC	35,485	36,918	48,454	3,913	3,972	3,985
FDP	32,352	36,514	51,616	4,048	4,111	4,130
DIB	83,072.82			4,096	4,219	4,250
MT	1600 (from FDP) 14,358 (from ISC, DIB)			3,864	3,864	3,864

CZK 1 = USD 0.048 as of 17th May 2021

In order to clarify and simplify the input costs of ISC and FDP health conditions, their mean value was used in the model. This value was calculated as the arithmetic mean of all three values corresponding to the respective treatment variants. In the case of ISC treatment, the mean value was set at CZK 40,286 (USD 1,923.24) and in the case of FDP treatment at CZK 40,161 (USD 1917.27).

### Utility values of health states

The utility values associated with the ISC, FDP and MT health states in the model were drawn from a Canadian study [13], published in 2007 at BMC Oral Health. The health utility of DIB is based on a study [25] published in the United Kingdom. The resulting utility values are shown in Table 4.

**Table 4:** Utility values in the Markov model

Health state	Utility value
ISC	74.75
FDP	71.47
DIB	59.19
MT	0.00

## Outcomes of Markov decision model

**Fig. 3:**
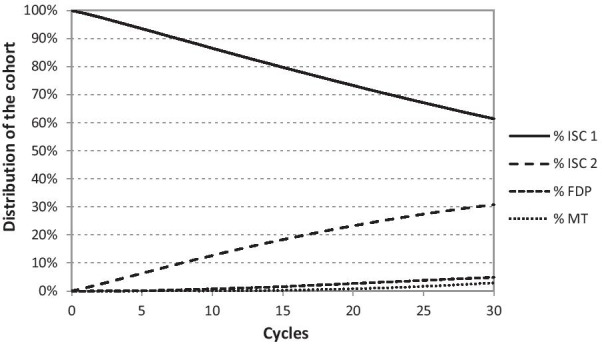
Distribution of cohort in individual cycles (ISC) appearing in the Markov tree model over a time horizon of 30 years. First implant-supported single crown (ISC 1), second implant-supported single crown (ISC 2), three-unit fixed dental prosthesis (FDP), missing tooth (MT)

**Fig. 4:**
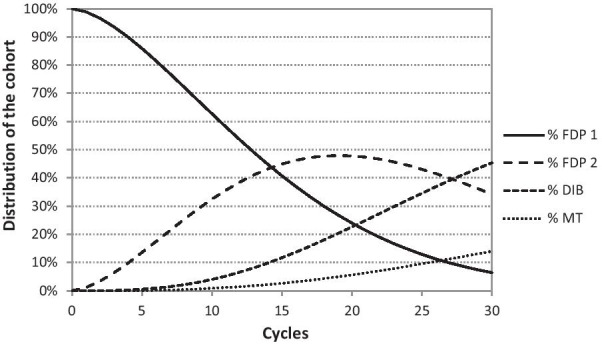
Distribution of cohort in individual cycles (FDP) appearing in the Markov tree model over a time horizon of 30 years. First three-unit fixed dental prosthesis (FDP 1), second three-unit fixed dental prosthesis (FDP 2), double implant-supported bridge (DIB), missing tooth (MT)

The considered cohort in the model in individual cycles, depending on the transition probabilities and probabilities of survival of the given replacement, gradually spreads to individual health states in the entire Markov tree. The proportions of the cohort appearing in both Markov tree models within the 30-year time horizon are shown in the following charts (Figs. 3, 4).

**Fig. 5:**
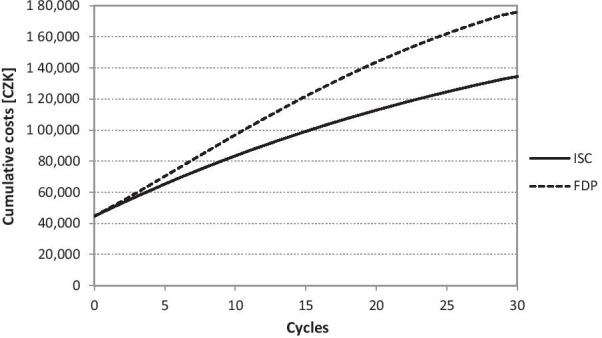
Cumulative costs during the 30 years (CZK 1 = USD 0.048 as of 17^th^ May 2021). Implant-supported single crown (ISC), three-unit fixed dental prosthesis (FDP)

**Fig. 6:**
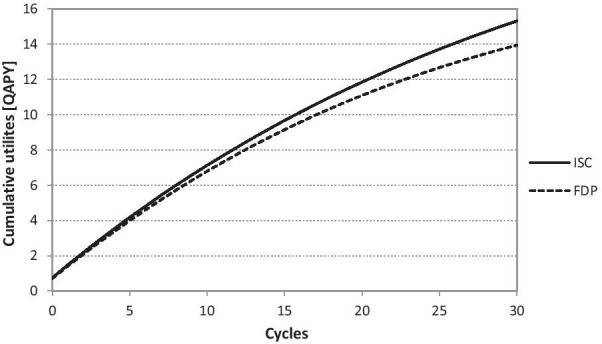
Cumulative utilities during 30 years. Implant-supported single crown (ISC), three-unit fixed dental prosthesis (FDP)

The distribution of the cohort is connected also with the division of costs and utility. The cumulative costs of the Markov model ISC correspond to CZK 134,514 (USD 6,421.64) after 30 years. The cumulative costs of the Markov model FDP correspond to CZK 175,923 (USD 8,398.49) after 30 years. The cumulative utility in the value of 12.57 QAPY corresponds to the Markov model ISC. The cumulative utility in the value of 11.32 QAPY corresponds to the Markov model FDP. The cumulative costs chart is presented in the Fig. 5 and the cumulative utilities chart is presented in Fig. 6.

**Fig. 7:**
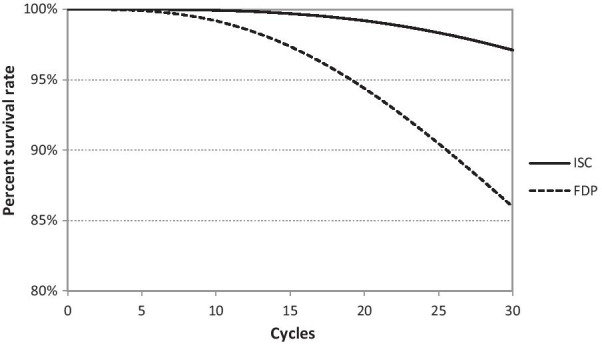
Survival curve over considered to the time horizon of 30 years. Implant-supported single crown (ISC), three-unit fixed dental prosthesis (FDP)

The evaluated modelled survival curves of individual treatment strategies over the entire considered time horizon are as follows (Fig. 7).

### Results of modelling cost-utility analysis

Cost-utility analysis modelling has shown that treatment with ISC appears to be the dominant intervention after 30 cycles of the model. In contrast, treatment with FDP in the model appears to be an absolutely dominated intervention. The final results are presented in Table 5. The value of the cost per unit of effect (CE) is CZK 8,787.57 (USD 419.52) in the case of ISC and CZK 12,628.84 (USD 602.90) in the case of FDP. The incremental cost-utility ratio (ICUR) is based on CZK -30,072.05 (USD -1,435.63). The result is also confirmed by the implementation of values within the incremental cost-utility plane, where the evaluated intervention moves in the lower right quadrant and is therefore defined as clearly cost-effective.

**Table 5:** Evaluation of CUA within a 30-year time horizon

Intervention	Costs (CZK)	Incremental costs (CZK)	Outcomes (QAPY)	Incremental outcomes (QAPY)	CE (CZK)	ICUR
ISC	134,513.74	0	15.31	0	8,787.57	0
FDP	175,923.11	− 41,409.37	13.93	1.38	12,628.84	− 30,072.05

CZK 1 = USD 0.048 as of 17th May 2021

### Sensitivity analysis

**Fig. 8:**
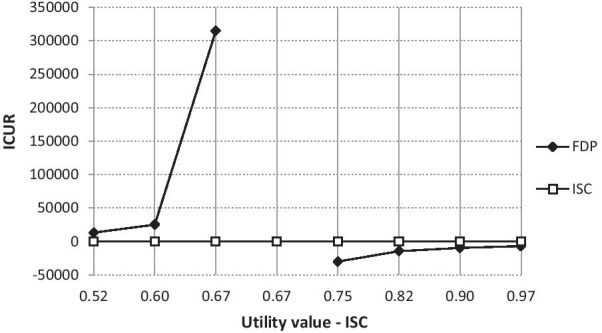
Sensitivity analysis—utility value (ISC; CZK 1 = USD 0.048 as of 17^th^ May 2021). Implant-supported single crown (ISC), three-unit fixed dental prosthesis (FDP), incremental cost-utility ratio (ICUR)

**Fig. 9:**
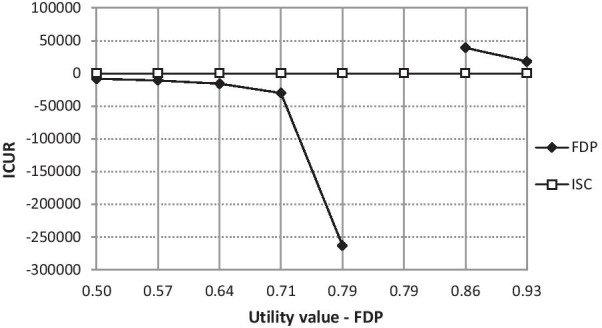
Sensitivity analysis—utility (FDP; CZK 1 = USD 0.048 as of 17^th^ May 2021). Implant-supported single crown (ISC), three-unit fixed dental prosthesis (FDP), incremental cost-utility ratio (ICUR)

Sensitivity analysis showed that the relationship of dominance of interventions by most changes was not disturbed. Changes in the perception of the dominance of interventions occur only if the values of utilities vary in the interval of ± 30%. The situation is presented in the graphs of Figs. 8 and 9. In both cases, the ICUR is transformed from positive to negative values, respectively from negative values to positive values. In the case of varying the values of the ISC treatment utilities, it is obvious that if the value of the ISC treatment utility is equal to, for example, 0.52325 (change -30%), the ICUR will correspond to a positive value of CZK 13,041.57 (USD 622.60). According to the incremental cost-utility plane interpretation, this means that if the ICUR value were greater than the willingness-to-pay (WTP) threshold, the evaluated intervention would be cost-effective in such conditions. Conversely, if the WTP exceeded this ICUR value, the evaluated ISC intervention would not be cost-effective. The highest ICUR value is reported by the ISC utility at 0.67275. The break is the utility value corresponding to 0.74078. From this value, the evaluated intervention is again clearly cost-effective, i.e. dominant.

The scenario is similar in the case of changing the utility values for FDP. Here, however, the reverse sequence occurs. Up to the value of the utility expressed exactly 0.8487576, the evaluated intervention is clearly cost-effective. From this value onwards, the ICUR is in positive numbers and the WTP threshold decides on the unambiguousness of cost-effectiveness.

**Fig. 10:**
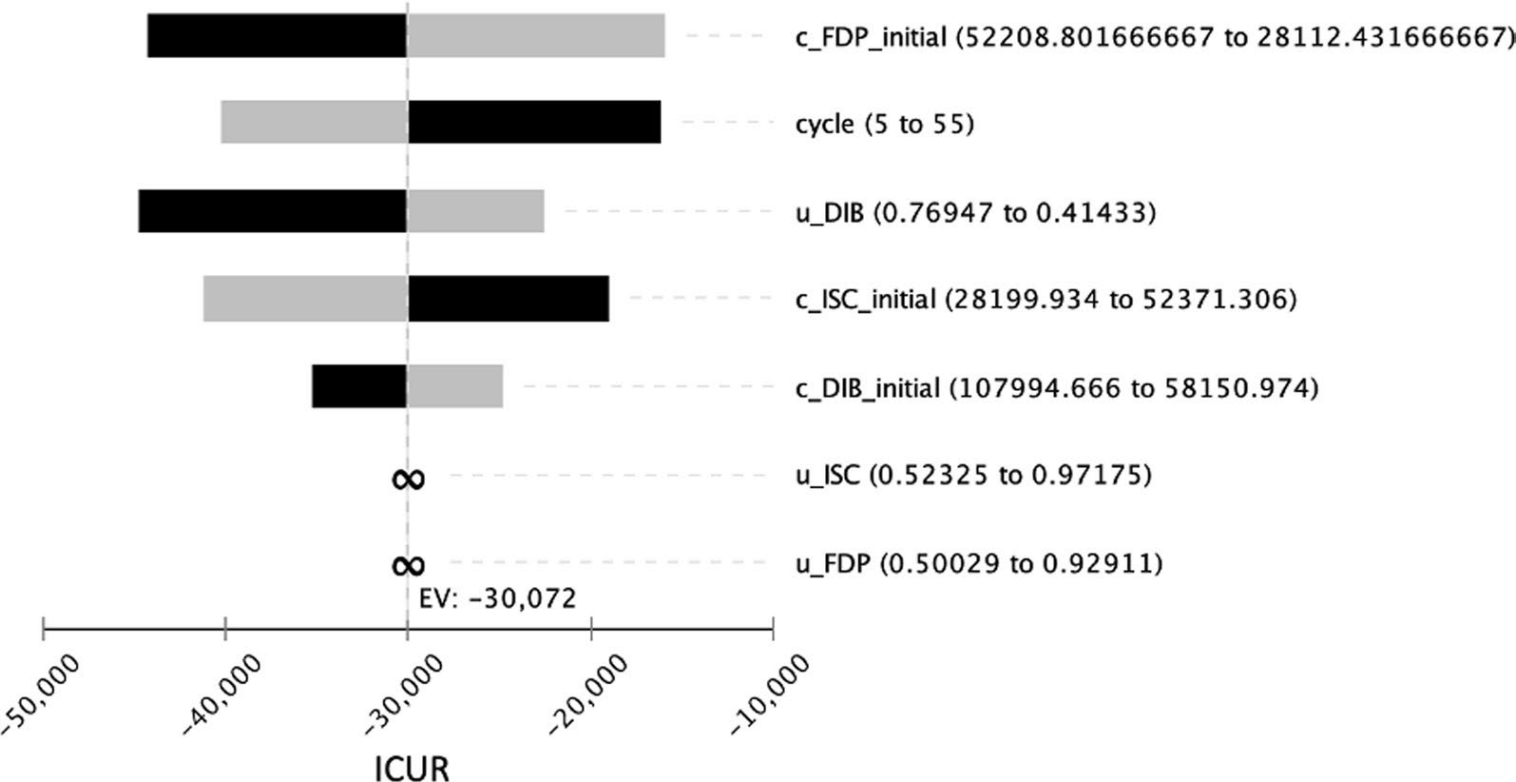
The tornado diagram shows the relationship of intervention dominance by changes in inputs in the interval of ± 30% (CZK 1 = USD 0.048 as of 17^th^ May 2021). Initial costs of three-unit fixed dental prosthesis (c_FDP_initital), utility of double implant-supported bridge (u_DIB), initial costs of implant-supported single crown (c_ISC_initial), initial costs of double implant-supported bridge (c_DIB_initial), utility of implant-supported single crown (u_ISC), utility of three-unit fixed dental prosthesis (u_FDP), incremental cost-utility ratio (ICUR)

From the results of the Tornado diagram, it is clear that the values of input costs for FDP treatment show the highest sensitivity. The graph (Fig. 10) shows the variance of the values of all variables depending on the changes in the parameters in the interval ± 30%. The input costs for the treatment of FDP are varied between CZK 28,112.43 (USD 1342.02) and CZK 52,208.80 (USD 2492.32) and thus correspond to the variance of ICUR values exactly between CZK −44,238 (USD −2111.81) and CZK -15,906 (USD -759.31).

## Discussion

The study focuses on the replacement of one lost tooth in the molar area, which in terms of the frequency of occurrence of missing teeth is among the most common [26, 27]. The study by Mack et al. [28] proves that the first molar is the most frequently missing tooth in the population between 20 and 74 years of age. Most published foreign cost-effectiveness studies [1, 5, 7–13, 15] used FDP for comparison with implant treatment. Endodontic treatment [8, 12, 29] or treatment with a removable prosthesis [13] was also often chosen as a comparator. Partial removable prosthesis was not considered in this study as it appears to be the solution with the lowest quality-adjusted tooth years (QATY) based on Oral Health-related Quality of Life (OHRQoL) measurements [13]. Indirect costs were included in the total cost of the initial treatment phase. In contrast, the study by Bouchard et al. [9] took into account only direct costs. The impact of the inclusion of indirect costs and direct non-medical costs on the total costs was assessed through a sensitivity analysis.

The factor that mainly affects the amount of average direct medical costs for the initial phase of treatment is the cooperation of the workplace with the health insurance company. The results of this study show that the differences in costs based on the factor of cooperation with the health insurance company are more pronounced under the assumption of the choice of FDP. We can therefore say that if a patient chooses FDP and a clinic that does not have a contract with health insurance company, he will pay on average CZK 9,403.22 (USD 448.89) more than at a clinic that cooperate with the patient's health insurance company. However, it is important to mention that this result is based on an assumption that does not take into account the selected treatment variant, it is a simple arithmetic average for a given treatment alternative. This difference is only CZK 2,128.77 (USD 101.62) for ISC. It is clear from the results that the costs of the initial phase of implant treatment are not significantly affected by the factor of the clinic's cooperation with the health insurance company. The result confirms the presumption of non-participation of the health insurance company in reimbursement of implant treatment and is in accordance with Alison's study [30]. Bouchard et al. [9] states that even in France, the participation of health insurance company in the payment for ISC is negligible, in contrast to the payment for FDP. The situation is similar in Japan where implant treatment is reimbursed only if the patient loses or damages his jaw due to illness or accident [5].

A limitation of this study is the use of the QAPY parameter instead of the QALY parameter. However, in the field of dentistry, the presentation of benefits through QALY or Life Year Gained is relatively difficult [4].

To strengthen the level of evidence, it would be appropriate to obtain data from more than 13 Prague clinics and at best scenario to compile a set of clinics from non-Prague workplaces where the cost of dental care may be lower. Furthermore, it would be appropriate to consider a treatment that does not involve the replacement of only one tooth, but several teeth, or, for example, a dental material other than ceramics (although ceramics are now considered a material with the required level of quality and aesthetics) [31].

The objectives of the study were based on the assumption that treatment of single tooth loss with an ISC is generally more expensive than treatment with FDP [6–8]. This primary assumption led to the use of a method of modelling using Markov models which should identify a value representing the equality of the cumulative costs of both treatments, the so-called turning point [6]. However, this primary assumption was not confirmed during the data analysis. It seems that in the CUA model this could reflect the use of the mean value of the input costs of both treatments. However, additional analysis revealed that even under the assumption of using precisely calculated input costs of individual treatment variants, there is ultimately no change in the CUA result. The reason of non-fulfilment of the primary assumption of work (higher initial costs for implant treatment) can also be the consideration of ceramics as a material for the production of FDP and also ISC [6–8]. However, the advantages of ceramics have already been discussed above [31]. Another reason may be the need for endodontic treatment of abutment teeth which, according to information from the panel of experts, is very often a condition for the deployment of a three-unit fixed dental prosthesis. In joint interviews with the doctors of the expert team, it was even found that many doctors consider this treatment to be automatic before using FDP. However, it must be said that this is a treatment which, based on the data obtained from dental clinics in Prague, will cost an average of CZK 15,731.31 (USD 750,97) with a standard deviation of CZK 4,412.32 (USD 210,63) (endodontics of both abutment teeth is considered).

The structure of the model takes into account not only the input costs of treatment, but also the annual costs that the patient will pay for solving possible complications and for inspections and preventive visits to the dentist and dental hygienist [1]. The probabilities of the occurrence of these events were obtained from a systematic search in literature [32]. The total costs associated with FDP in the model are generally higher than the cost of implant treatment. The same conclusion was reached by a study [14], which evaluates ISC even with inclusion of indirect costs (due to the higher number of visits per implant treatment) as cheaper treatment. Another study [10] even provides evidence of higher total costs of initial FDP compared to ISC because of laboratory costs. Chun et al. [1] demonstrate that although the direct input costs of ISC are initially higher compared to FDP, after a 10-year time horizon, ISC becomes less expensive in terms of direct treatment costs.

Within the model, it was necessary to solve the issue of data transferability. Data on transition probabilities were obtained from individual studies found in systematic search; more accurate data could be obtained from a metanalysis, but this type of study is not suitable for fitting the Kaplan–Meier survival curve. Data on utilities of individual health conditions were transferred to the model. The source of utilities for the condition of implant treatment, fixed bridge and the condition of the missing tooth was a Canadian study [13]. The primary requirement in selecting the study was its complexity to provide utility values for all health conditions considered in the model, it’s appropriate year of publication (not older than 15 years), and finally the appropriate age of the target population (50 years). A Canadian study [13] meets all primary requirements except for the condition of gaining values for all health conditions considered in the model. No other such study was found. In order to obtain data on the utility of DIB treatment, which was not addressed in a Canadian study [13], another study [25] had to be used. This was a study directly from the United Kingdom, which considered the same target population and publication was not older than 15 years [25]. To express utility value for DIB state and to compare it with the utility values from the Canadian study [13], this study [25] was chosen for several other reasons. It is a study [25] similar to the Canadian study [13]: including comparable options in different treatment options; study that uses one of the recommended valuation methods (Visual Analogue Scale) to express the utility; study with a sufficiently large number of patients.

As the time horizon of this clinical-economic model was longer than one year, the costs and benefits had to be discounted at the recommended discount rate, which according to Czech Pharmacoeconomic Society [2] is 3%. The recommendation of the State Institute for Drug Control [33] is the use of 0% and 5% discount rates in the sensitivity analysis.

Modelling of CUA confirmed that after a 30-year time horizon for the use of a prosthesis in the oral cavity, FDP appears to be not clearly cost-effective. The result of CUA modelling coincides with the results of several foreign studies [10, 11, 15], which also used modelling using Markov models with a longer time frame for more accurate and flexible results. The incremental cost-utility ratio in this study is -30 072 CZK (USD -1,435.56). The evaluated intervention thus became clearly cost-effective. A French study [9] came to the same conclusion and the authors argue that the unambiguity of the result should lead to the first place of this treatment in all European countries. However, it should be noted that only a 20-year time horizon was considered in the study. Another study [7], which considers a 10-year time horizon, has the opposite results and ISC is dominated intervention. However, sensitivity analysis states that for ISC to become the dominant intervention, it is sufficient that the cost of ISC treatment is reduced by 20%.

The model is transferable between other countries, it is possible to make specific adjustments typical for a given country and to use collected inputs. In contrast to the clinical outcomes and the probabilities used, it is not possible to consider the cost side of the study. Although proportionally we can assume the same cost results and thus the same results for more developed countries with a similar health care system as in the Czech Republic.

## Conclusion

Clinical-economic evaluations provide physicians as well as patients with important information on the cost-effectiveness of medical technologies. However, such evaluations are still rather rare in dentistry. In addition, there is a significant need for use of appropriate methodology for a correct estimate of cost-effectiveness. This work brings new knowledge about the cost-effectiveness of ISC in the conditions of the Czech healthcare system. Although there is speculation in dentistry that ISC is more expensive than FDP, and this fact was the original premise of this study, the results speak differently. Based on the modelling, it was found that over the entire time horizon of 30 years, ISC shows significantly lower cumulative costs than FDP treatment and brings a higher effect. In addition, the conventional solution of replacing one missing tooth with FDP exposes abutment teeth to many biological and technical problems. ISC is therefore a suitable first-choice solution for adult patients who has one missing tooth in the molar area and are willing to undergo surgery. For them, the treatment becomes cost-effective.

## Data Availability

The datasets generated during and analysed during the current study are in manuscript or not publicly available because they are data from private entities that have consented to publication only in the form of final results that are in manuscript or are available from the corresponding author on reasonable request.

## Abbreviations

CEA: Cost-effectiveness analysis
CSO: Czech Statistical Office
CUA: Cost-utility analysis
DIB: Double implant-supported bridge
FDP: Three-unit fixed dental prosthesis
ICUR: Incremental cost-utility ratio
ISC: Implant-supported single crown
MT: Missing tooth
OHRQoL: Oral Health related Quality of Life
QALY: Quality-adjusted life year
QAPY: Quality-adjusted prosthesis years
QATY: Quality-adjusted tooth years
WTP: Willingness to pay
